# Comparative Genomic and Functional Characterization of Two Lytic Bacteriophages Against Antimicrobial-Resistant *Escherichia coli*

**DOI:** 10.3390/antibiotics15060563

**Published:** 2026-06-01

**Authors:** Tasnime A. Abdo Ahmad, Zahraa Shokor, Hadi Hussein, Lynn El Haddad, Roy F. Chemaly, Ghassan M. Matar, Esber S. Saba

**Affiliations:** 1 Department of Experimental Pathology, Immunology & Microbiology, Faculty of Medicine, American University of Beirut, Beirut 1107 2020, Lebanon; taa53@mail.aub.edu (T.A.A.A.); zas34@mail.aub.edu (Z.S.); hh161@aub.edu.lb (H.H.); gmatar@aub.edu.lb (G.M.M.); 2 Department of Infectious Diseases, Infection Control, and Employee Health, University of Texas MD Anderson Cancer Center, Houston, TX 77030, USA; lel2@mdanderson.org (L.E.H.); rfchemaly@mdanderson.org (R.F.C.); 3 Center of Infectious Diseases Research, Faculty of Medicine, American University of Beirut, Beirut 1107 2020, Lebanon; 4 WHO CC for Reference & Research on Bacterial Pathogens, Faculty of Medicine, American University of Beirut, Beirut 1107 2020, Lebanon

**Keywords:** AMR, *Escherichia coli*, bacteriophage therapy, lytic bacteriophages, biofilm disruption, host–phage interactions, T4-like phages, CRISPR systems, adsorption mechanisms

## Abstract

Background/Objectives: Antimicrobial resistance (AMR) in *Escherichia coli* is a growing public health concern, particularly in regions affected by environmental contamination and poor wastewater management. Data on locally isolated *E. coli*-targeting phages in Lebanon remain limited. This study aimed to isolate, characterize, and evaluate two lytic bacteriophages against AMR *E. coli*. Methods: Two phages, EPIMAM01 (gb:PQ493298) and EPIMRB01 (gb:PQ657784), were isolated from untreated sewage in Beirut using *E. coli* ATCC 25922. Characterization included double-layer agar assays, one-step growth analysis, and stability testing across temperature and pH ranges. Bacteriolytic activity was assessed in planktonic cultures and preformed biofilms. Host range and efficiency of plating (EOP) were evaluated using clinical isolates. Whole-genome sequencing and comparative analyses were performed. Results: Both phages produced clear plaques and showed a latent period of ~40 min. EPIMAM01 had a higher estimated burst size (140 PFU/infected cell) than EPIMRB01 (75 PFU/infected cell). Both phages remained stable between 4–50 °C and within a pH range of 5–10 but showed marked loss of activity at temperatures ≥60 °C and pH ≤3 or ≥12. EPIMAM01 effectively inhibited planktonic growth of *E. coli* ATCC 25922, whereas EPIMRB01 showed stronger biofilm-disrupting activity against preformed *E. coli* biofilms. Both phages lysed several of the 17 tested clinical *E. coli* isolates. Comparative analyses of gene presence/absence patterns, bacterial defense systems, and adsorption phenotypes among the tested *E. coli* strains identified *mlaA*, *ydcQ*, and ompD-2 as candidate adsorption-associated genes and suggested CRISPR systems may reduce susceptibility. Genomic analysis classified both phages as T4-like phages lacking lysogeny, virulence, or AMR genes. Conclusions: EPIMAM01 and EPIMRB01 are lytic phages with complementary antimicrobial properties, supporting their potential for further development as AMR control agents.

## 1. Introduction

*Escherichia coli* is a Gram-negative bacterium that commonly inhabits the human gastrointestinal tract as a commensal organism, yet certain strains can act as important opportunistic pathogens. It is a frequent cause of urinary tract infections, pneumonia, bacteremia, and other serious infections, particularly when multidrug-resistant (MDR) strains are involved [1]. The increasing spread of AMR in *E. coli* has become a major public health concern worldwide, reducing treatment options and increasing the burden of infection.

Environmental dissemination further contributes to the spread of resistant *E. coli*. Because the organism is shed in human and animal feces, contaminated water systems can act as important reservoirs and transmission routes. In Lebanon, several studies have highlighted significant fecal contamination of rivers and water systems, including widespread detection of *E. coli* and a substantial proportion of MDR isolates [2]. Although river water is not typically consumed directly, it contributes to environmental circulation through irrigation, coastal discharge, and broader ecological spread. These conditions make the search for locally relevant antibacterial alternatives especially important [3].

Bacteriophages are viruses that infect bacteria and are increasingly being revisited as potential tools to combat AMR. Of particular interest are lytic phages, which bind to specific bacterial receptors, inject their genetic material, replicate within the host, and ultimately lyse the infected cell. Their host specificity, self-amplifying nature, and antibacterial activity make them attractive candidates for therapeutic and biocontrol applications. In addition to lytic efficiency, properties such as adsorption dynamics, host range, environmental stability, and biofilm-disruption capacity are important determinants of phage therapeutic potential. Understanding the genomic basis underlying these phenotypic traits may help improve phage selection and future engineering strategies [4].

Despite growing international interest in phage therapy, phages active against *E. coli* in Lebanon remain insufficiently characterized. This gap limits the development of locally informed phage-based strategies against MDR *E. coli*. Accordingly, the aim of this study was to isolate and comparatively characterize two lytic phages active against *E. coli* from untreated wastewater in Beirut, with emphasis on their biological properties, genomic features, host range, and associations with bacterial defense systems. We hypothesized that these wastewater-derived phages would display distinct functional and genomic characteristics that could help explain differences in antibacterial activity and host range.

## 2. Results

### 2.1. Isolation of Escherichia Phages

**Figure 1:**
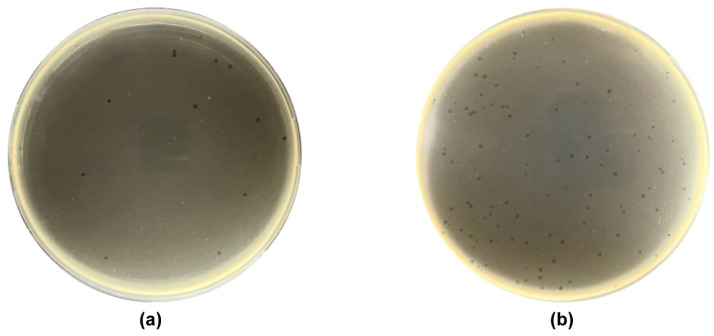
Representative plaque morphology of bacteriophages EPIMAM01 and EPIMRB01 on *E. coli* ATCC 25922 lawns. (**a**) Plaques formed by EPIMAM01. (**b**) Plaques formed by EPIMRB01.

EPIMAM01 and EPIMRB01 were isolated against *E. coli* ATCC 25922 from untreated wastewater sources at Ain El-Mreiseh and Ramlet El-Bayda in Beirut, Lebanon. Following filtration and enrichment steps, phage-containing supernatants were mixed with exponentially growing *E. coli* ATCC 25922 cultures and overlaid on agar plates. After incubation, both phages produced distinct clear, circular plaques, indicating efficient lysis of the bacterial host. The appearance of well-defined, transparent lysis zones was consistent with a lytic phenotype, which was later supported by genomic analysis showing no lysogeny-associated genes (Figure 1a,b).

### 2.2. Adsorption Kinetics and One-Step Growth Analysis of EPIMAM01 and EPIMRB01

**Figure 2:**
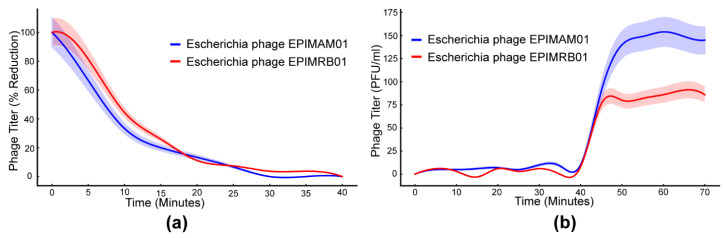
Adsorption Rate and One-Step Growth Curves of EPIMAM 01 and EPIMRB 01. (**a**) Adsorption kinetics of EPIMAM01 and EPIMRB01 over 40 min. (**b**) One-step growth curves of EPIMAM01 and EPIMRB01. Shaded areas represent standard deviation from three independent experiments.

The adsorption rates of EPIMAM01 and EPIMRB01 were evaluated against *E. coli* ATCC 25922 over a 40-min time course (Figure 2a). Both phages exhibited rapid initial adsorption within the first 10 min, during which more than 50% of free phage particles had attached to the bacterial surface. EPIMAM01 demonstrated a slightly higher adsorption efficiency compared to EPIMRB01, with both phages achieving nearly complete adsorption (~90%) by 20 min. This indicates strong affinity and rapid binding capability to the *E. coli* host, which is critical for efficient infection initiation. The replication dynamics were further examined using one-step growth experiments (Figure 2b).

Both phages displayed a latent period of approximately 40 min, representing the time required for intracellular replication, assembly, and maturation before host cell lysis. Following the latent phase, a marked increase in phage titers was observed, reflecting successful release of progeny virions.

Using the initial bacterial concentration and the experimentally observed adsorption efficiency, the burst size was estimated to be approximately 140 PFU per infected cell for EPIMAM01 and approximately 75 PFU per infected cell for EPIMRB01.

These results indicate that EPIMAM01 produces a greater number of progeny phage particles per infected cell, potentially explaining its stronger lytic activity observed in plaque and inhibition assays.

Together, these results highlight the efficient adsorption and productive replication cycles of both EPIMAM01 and EPIMRB01 against *E. coli* ATCC 25922, supporting their further evaluation as antibacterial phages for therapeutic or biocontrol research.

### 2.3. Inhibition of E. coli ATCC 25922 Growth by EPIMAM01 and EPIMRB01 at Different MOIs

**Figure 3:**
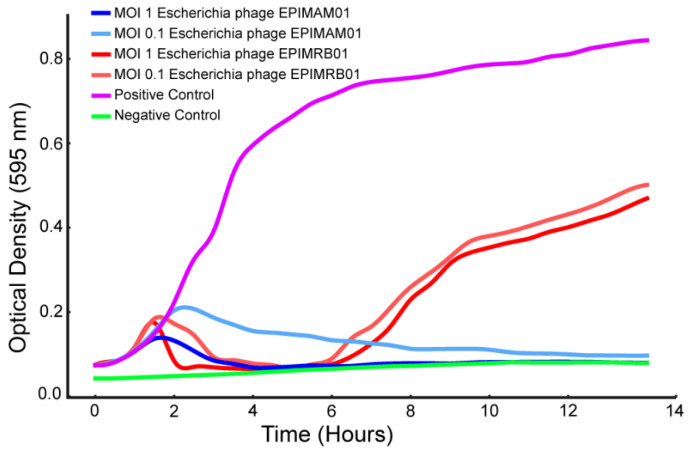
Inhibition of *Escherichia coli* ATCC 25922 growth by phages EPIMAM01 and EPIMRB01 at different multiplicities of infection (MOIs). Bacterial growth was monitored by measuring optical density at 600 nm over 14 h. Phages were tested at MOI 1 and MOI 0.1. Positive control: untreated *E. coli* culture. Negative control: sterile medium.

The lytic activity of phages EPIMAM01 and EPIMRB01 against *E. coli* ATCC 25922 was evaluated by monitoring bacterial growth dynamics over 14 h at multiplicities of infection (MOI) of 1 and 0.1 (Figure 3). Bacterial growth was assessed by measuring optical density (OD) at 600 nm. In the positive control group (untreated *E. coli* culture), a typical exponential growth curve was observed, reaching an OD of approximately 0.9 after 14 h. In contrast, the negative control (medium only) showed no increase in OD, confirming the absence of bacterial contamination.

Phage EPIMAM01 exhibited strong inhibitory effects at both MOI values. At MOI 1, complete suppression of bacterial growth was observed throughout the experiment, as indicated by a consistently low OD. Even at a lower MOI of 0.1, EPIMAM01 delayed bacterial regrowth, maintaining OD values below 0.2 for the entire period. This suggests that EPIMAM01 possesses potent lytic activity and can effectively control bacterial populations even at reduced phage concentrations.

EPIMRB01 also suppressed bacterial growth, although to a lesser extent than EPIMAM01. At MOI 1, EPIMRB01 initially reduced OD values, but partial regrowth was observed after approximately 5 h, indicating possible emergence of resistant bacterial subpopulations or reduced phage stability. At MOI 0.1, EPIMRB01 delayed growth but was less effective, with OD values gradually increasing after 4 h and reaching higher levels compared to EPIMAM01-treated groups.

Overall, these results demonstrate that both phages can inhibit *E. coli* growth in vitro, with EPIMAM01 showing superior and more sustained antibacterial activity. The pronounced difference in performance at lower MOI further highlights EPIMAM01’s potential as a robust candidate for therapeutic or biocontrol applications.

### 2.4. Stability of EPIMAM01 and EPIMRB01 Under Different Temperature and pH Conditions

**Figure 4:**
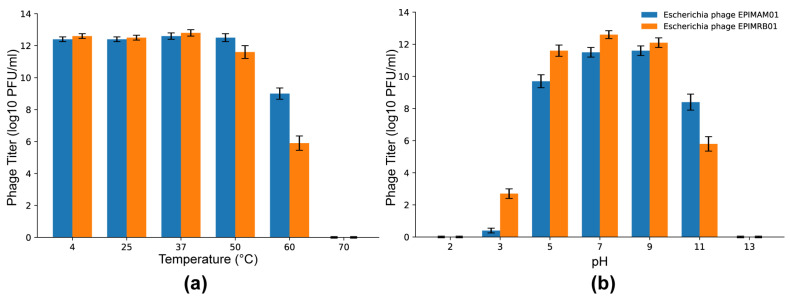
Stability of Escherichia phages EPIMAM01 and EPIMRB01 under different temperature and pH conditions. Phage titers (log10 PFU/mL) following incubation under different temperature conditions (**a**) and pH values (**b**). Bars represent mean values from three independent experiments and error bars indicate standard deviations.

The stability profiles of phages EPIMAM01 and EPIMRB01 were assessed across a range of temperatures and pH values to evaluate their robustness under varying environmental conditions (Figure 4).

For thermal stability, both phages maintained high titers (approximately 10^12^–10^13^ PFU/mL) following incubation at temperatures ranging from 4 °C to 50 °C. This indicates strong resistance to moderate heat, supporting their potential application in diverse environmental or therapeutic settings. A sharp decline in phage titers was observed at temperatures above 60 °C, with complete inactivation detected at 70 °C, highlighting their heat sensitivity at higher extremes. Notably, EPIMAM01 showed slightly better stability than EPIMRB01 at intermediate temperatures (40–50 °C), suggesting marginal differences in capsid stability or structural protein resilience.

Regarding pH stability, both phages demonstrated optimal stability in the pH range of 5 to 10. Titers remained high within this interval, indicating their capacity to withstand physiological and slightly acidic or alkaline conditions. EPIMRB01 exhibited broader pH tolerance, maintaining higher titers even at pH 4 and pH 11, compared to EPIMAM01, which showed a steeper decline outside the optimal range. Extreme acidic (pH ≤ 3) and alkaline (pH ≥ 12) conditions led to complete loss of phage activity, consistent with expected denaturation and structural degradation under harsh pH stress.

Overall, these results confirm that both EPIMAM01 and EPIMRB01 possess good thermal and pH stability within moderate environmental ranges, supporting their stability under moderate environmental conditions relevant to storage and downstream application.

### 2.5. Biofilm Disruption Activity of EPIMAM01 and EPIMRB01

**Figure 5:**
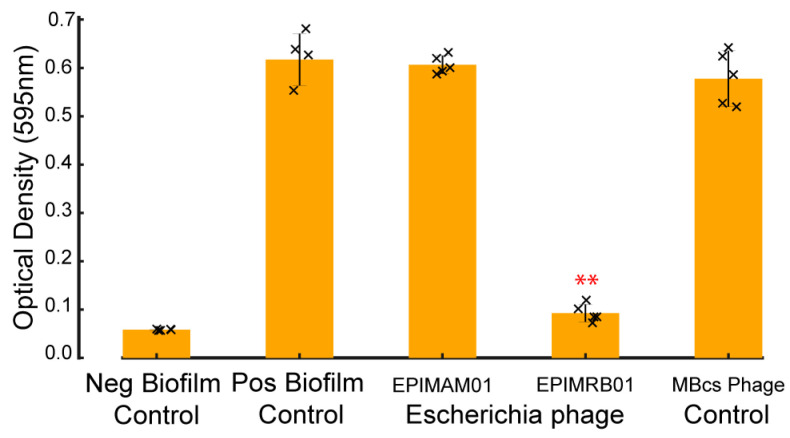
Disruption of *Escherichia coli* ATCC 25922 biofilms by phages EPIMAM01 and EPIMRB01. Biofilm biomass was quantified by crystal violet staining and measuring optical density at 600 nm. The negative biofilm control (Neg Biofilm Control) had only sterile medium. The positive biofilm control (Pos Biofilm Control) had only untreated *E. coli* biofilm with sterile medium. The MBcs phage control (targeting *Salmonella* spp.) as a non-*E. coli* phage control. Orange bars represented mean OD595 values, while error bars represented standard deviations from at least five independent experiments, with individual data points shown as crosses. Statistical significance was determined by one-way ANOVA followed by post hoc multiple-comparison testing. **: *p* < 0.1.

The ability of *Escherichia* phages EPIMAM01 and EPIMRB01 to disrupt pre-formed *E. coli* ATCC 25922 biofilms was evaluated using a crystal violet staining assay, with optical density measured at 600 nm to quantify biofilm biomass (Figure 5). As expected, the negative biofilm control (sterile medium) showed minimal absorbance, confirming the absence of biofilm formation. In contrast, the positive biofilm control (untreated *E. coli* culture) exhibited high optical density (~0.6), reflecting robust biofilm development.

Treatment with EPIMAM01 did not result in a significant reduction in biofilm biomass compared to the positive control, suggesting limited ability of this phage to penetrate or degrade established biofilms under the tested conditions. In contrast, EPIMRB01 resulted in a statistically significant reduction in biofilm biomass, as indicated by a substantial decrease in optical density to approximately 0.1 (*p* < 0.01). This reduction suggests strong biofilm-disrupting activity under the tested conditions, although the underlying mechanism remains to be determined. A control phage targeting *Salmonella* (MBcs phage) did not impact *E. coli* biofilm, confirming the specificity of the tested *Escherichia* phages.

Overall, these results highlight EPIMRB01 as a promising candidate for targeting and reducing *E. coli* biofilms, while EPIMAM01 appears to be more effective against planktonic cells rather than mature biofilm structures.

### 2.6. Genomic Characterization and Comparative Analysis of EPIMAM01 and EPIMRB01

**Figure 6:**
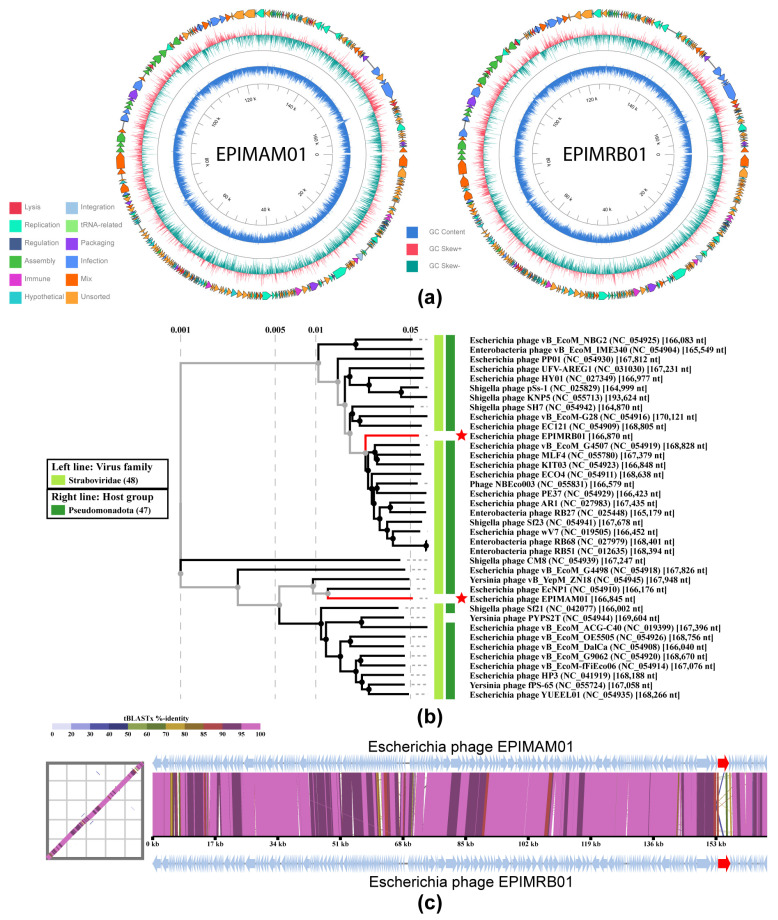
Comparative genomic analysis of bacteriophages EPIMAM01 and EPIMRB01. (**a**) Circular genome maps showing predicted coding sequences, tRNA genes, GC content, and GC skew. (**b**) Whole-genome phylogenetic tree of EPIMAM01, EPIMRB01, and related reference phages. Red stars indicate EPIMAM01 and EPIMRB01. Colored bars indicate virus family and host group. (**c**) Pairwise whole-genome alignment between EPIMAM01 and EPIMRB01 showing homologous locally collinear blocks represented in purple.

The genomic relatedness and evolutionary positioning of phages EPIMAM01 and EPIMRB01 were investigated using circular genome visualization, whole-genome phylogenetic analysis, and pairwise genome alignment (Figure 6). Circular genome maps showed that both phages possess similarly organized genomes with comparable distributions of predicted functional categories, including genes involved in replication, regulation, assembly, packaging, and lysis, together with similar GC content and GC skew profiles (Figure 6a). These overall similarities are consistent with a conserved genomic architecture.

Whole-genome phylogenetic analysis placed both EPIMAM01 and EPIMRB01 within a cluster of closely related *Escherichia* phages belonging to the family *Straboviridae* (Figure 6b). Although the two phages grouped within the same broader lineage and showed close relatedness to previously described T4-like *Escherichia* phages, they occupied distinct positions within the tree, as indicated by the red stars. This pattern supports a shared evolutionary background while also indicating that EPIMAM01 and EPIMRB01 are not identical and likely represent closely related but distinct genomes.

To further evaluate genomic relatedness and taxonomic classification, pairwise intergenomic similarity analysis was performed using VIRIDIC against the closest related reference phages (Supplementary Figure S1). The analysis demonstrated that both EPIMAM01 and EPIMRB01 clustered with T4-like *Escherichia* phages and showed high genomic similarity to related reference genomes (Supplementary Figure S1). EPIMAM01 exhibited pairwise intergenomic similarity values ranging from approximately 93.7–94.3%, whereas EPIMRB01 showed values ranging from approximately 92.3–94.2%. These values remained below the established species demarcation threshold of 95% intergenomic similarity, supporting the classification of EPIMAM01 and EPIMRB01 within the broader T4-like phage group while also highlighting their genomic distinctiveness relative to previously described phages.

Pairwise whole-genome alignment further supported this relationship by revealing extensive sequence conservation and a high degree of synteny across most of the genome length (Figure 6c). The alignment was dominated by large-conserved regions, indicating that the two phages share a common genomic backbone. However, localized regions of divergence were also evident, particularly toward the terminal portion of the genome, where sequence conservation was reduced. A detailed comparison of this divergent terminal region, including tail fiber-associated genes, is shown in Supplementary Figure S2. This variable region appears to include genes associated with host-recognition functions, including tail fiber-associated genes, which may contribute to differences in host interaction and infection behavior between the two phages.

To further characterize the major divergent terminal region observed in the pairwise whole-genome alignment, the corresponding ORFs were extracted from the GenBank annotations of EPIMAM01 and EPIMRB01 and compared at the protein level (Supplementary Tables S1 and S2 and Supplementary Figure S2). The proximal long-tail fiber proteins were highly conserved, with EPIMAM01_00248 versus EPIMRB01_00248 sharing 93.8% amino-acid identity and EPIMAM01_00249 versus EPIMRB01_00249 sharing 95.4% identity. In contrast, the distal long-tail fiber-associated region was more divergent. EPIMAM01_00250 encoded a 216-aa long-tail fiber protein, whereas the corresponding EPIMRB01 region was annotated as two CDSs, EPIMRB01_00250 and EPIMRB01_00251, encoding 175-aa and 40-aa proteins, respectively; together, these proteins shared 62.6% global amino-acid identity with EPIMAM01_00250. The adjacent downstream proteins, EPIMAM01_00251 and EPIMRB01_00252, shared only 29.0% global amino-acid identity. Conserved-domain prediction using NCBI CDD identified a Phage_T4_gp36 domain in the distal long-tail fiber proteins of both phages and a Peptidase_S74 domain in both adjacent downstream proteins. Additional predicted domain content differed: EPIMAM01_00251 contained three pyocin_knob domain hits, whereas EPIMRB01_00252 contained a PHA00430 hit. These findings identify localized sequence and predicted domain-architecture divergence in a terminal tail fiber-associated region that may contribute to the distinct phenotypic behaviors of the two phages; this interpretation remains hypothesis-generating and requires functional validation.

Overall, these data show that EPIMAM01 and EPIMRB01 are closely related T4-like *Escherichia* phages with strongly conserved genome organization but with discrete genomic differences in specific regions that may underlie the phenotypic differences observed in downstream functional assays.

To further characterize the major divergent terminal region observed in the pairwise whole-genome alignment, the corresponding ORFs were extracted from the GenBank annotations of EPIMAM01 and EPIMRB01 and compared at the protein level (Supplementary Table S1 and Supplementary Figure S2). This region contained genes annotated as long-tail fiber proteins and adjacent hypothetical proteins. The long-tail fiber proximal subunit was highly conserved between EPIMAM01_00248 and EPIMRB01_00248, with 93.8% amino-acid identity over proteins of equal length (1289 aa). The adjacent long-tail fiber protein was also conserved between EPIMAM01_00249 and EPIMRB01_00249, with 95.4% identity over 371 aa. In contrast, greater divergence was observed in the distal tail fiber-associated region. EPIMAM01_00250 encoded a 216-aa long-tail fiber protein, whereas the corresponding region in EPIMRB01 was annotated as two long-tail fiber CDSs, EPIMRB01_00250 and EPIMRB01_00251, encoding 175 aa and 40 aa proteins, respectively. When EPIMRB01_00250 and EPIMRB01_00251 were analyzed together, they shared 62.6% global amino-acid identity with EPIMAM01_00250. The downstream hypothetical protein was even more divergent, with EPIMAM01_00251 and EPIMRB01_00252 sharing 29.0% global amino-acid identity. These findings indicate that, despite the high overall genomic similarity between the two phages, the terminal tail fiber-associated region contains localized sequence divergence that may contribute to differences in host interaction, bacteriolytic activity, and biofilm-disruption phenotype. This interpretation remains hypothesis-generating and requires experimental validation.

### 2.7. Host Range and Genomic Prediction of Bacterial Defense System and Candidate Adsorption-Associated Genes

**Figure 7:**
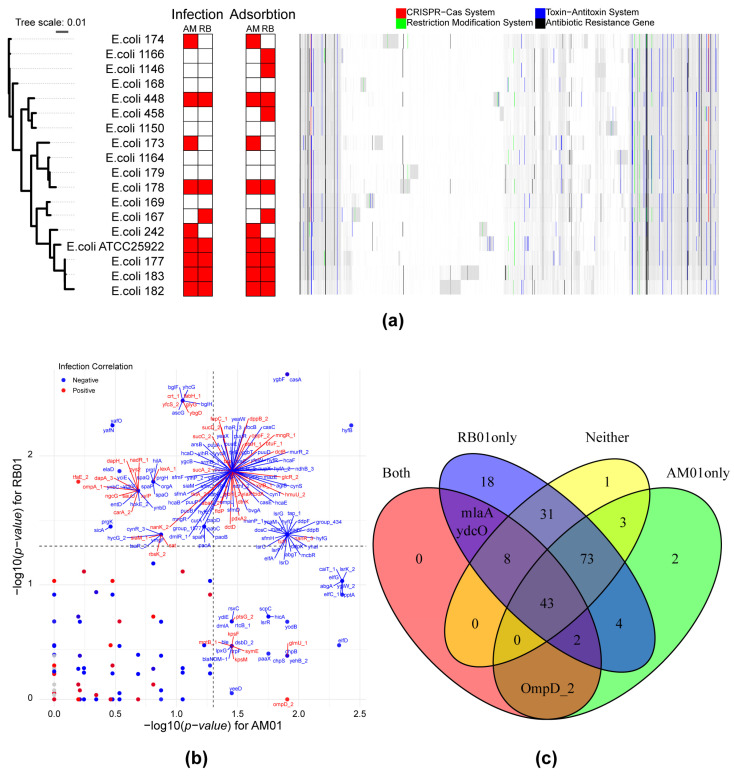
Host range, defense systems, gene associations, and receptor candidates in *E. coli* strains exposed to phages EPIMAM01 and EPIMRB01. (**a**) Dendrogram of *E. coli* strains based on whole-genome data with heatmaps showing infection and adsorption profiles together with bacterial defense systems. Red indicates presence/successful phenotype and white indicates absence. Defense systems are color-coded by category. (**b**) Scatter plot showing gene-level associations with phage infection outcomes represented as –log_10_(*p*-value) for EPIMAM01 (x-axis) and EPIMRB01 (y-axis). Points are colored according to correlation direction. (**c**) Venn diagram showing gene presence among *E. coli* strains grouped according to adsorption profiles toward EPIMAM01 and EPIMRB01.

To investigate the host range of EPIMAM01 and EPIMRB01, a panel of *E. coli* strains was tested [5] for susceptibility to infection and adsorption (Figure 7a). Phylogenetic analysis based on whole-genome data revealed clear clustering among the tested strains, reflecting their genetic diversity.

Adsorption assays demonstrated that both phages could attach to the majority of *E. coli* strains, as indicated by widespread red blocks in the adsorption columns. In this study, adsorption positivity reflected the ability of the phage to attach to the bacterial surface, whereas productive infection was defined by successful plaque formation and replication during the host range and EOP assays. Interestingly, a distinct subset of genes was identified in strains adsorbing EPIMRB01 However, differences emerged in subsequent infection assays. While EPIMAM01 successfully infected and replicated in a broader range of strains, EPIMRB01 exhibited a more restricted infection profile despite efficient adsorption. This suggests that some strains possess post-adsorption defense mechanisms preventing productive infection by EPIMRB01.

To explore potential genetic determinants of resistance, defense system profiling was performed across the *E. coli* panel (Figure 7a). This analysis revealed substantial heterogeneity in the distribution of anti-phage defense elements, including CRISPR-Cas systems (red), restriction–modification systems (green), and various toxin-antitoxin systems (blue). Notably, strains that resisted EPIMRB01 infection despite successful adsorption often harbored multiple or robust defense systems, suggesting a possible correlation between defense system abundance and phage resistance. In contrast, strains susceptible to both phages typically showed fewer or absent defense elements.

To further dissect genetic determinants influencing phage susceptibility, a comprehensive gene-level association analysis was performed using Fisher exact tests (Figure 7b). For each gene, the significance of its association with infection outcome was represented as –log_10_(*p*-value), plotted for EPIMAM01 (x-axis) and EPIMRB01 (y-axis). The scatter plot revealed several genes significantly correlated with resistance or susceptibility to one or both phages. Genes located in the upper right quadrant displayed strong associations with infection outcomes for both phages, suggesting shared genetic determinants affecting general phage defense mechanisms. In contrast, genes scattered along one axis but not the other indicated phage-specific associations. For example, genes with high –log_10_(*p*-value) values for EPIMRB01 but not EPIMAM01 (upper left quadrant) likely contribute to EPIMRB01-specific resistance or susceptibility profiles, and vice versa. Color coding further distinguished the directionality of the correlations: genes in red indicated a positive correlation with infection (potentially facilitating phage entry or replication), whereas genes in blue indicated a negative correlation (potentially mediating resistance). Notably, among the negatively correlated genes, two important examples emerged: *ygbF*, which encodes Cas2, a CRISPR-associated endoribonuclease involved in spacer acquisition, and *casA*, another CRISPR-associated gene. These findings suggest that CRISPR-mediated immunity plays a significant role in shaping strain-specific resistance profiles, especially against EPIMRB01. The overall distribution highlights a complex genetic landscape underlying phage-host interactions, with both shared and phage-specific factors contributing to differential infection outcomes. These insights provide a valuable foundation for exploring phage resistance mechanisms and inform the rational design of effective phage cocktails or engineered phages targeting MDR *E. coli* strains.

To identify candidate bacterial receptors mediating phage adsorption, a comparative gene presence/absence analysis was performed among *E. coli* strains grouped by their adsorption profiles to EPIMAM01 and EPIMRB01 (Figure 7c). The Venn diagram illustrates the distribution of genes potentially associated with surface-related proteins. A large core set of 159 genes was shared among strains not adsorbing to either phage, suggesting these genes are unlikely to be involved in phage binding.

Interestingly, a distinct subset of genes was identified in strains adsorbing EPIMRB01. Among these, *mlaA* and *ydcQ* were associated with strains showing adsorption to EPIMRB01 and may represent candidate genes linked to surface features relevant to adsorption. Likewise, ompD-2 emerged as a candidate gene associated with adsorption patterns involving EPIMAM01. These associations should be considered hypothesis-generating and require experimental validation before being interpreted as receptor determinants.

Functionally, EPIMAM01 exhibited a remarkable ability to replicate and kill all *E. coli* strains to which it successfully adsorbed, indicating that adsorption reliably reflected productive infection and the presence of a functional receptor. In contrast, EPIMRB01 did not consistently replicate in all strains it adsorbed to, supporting the presence of additional intracellular defense mechanisms beyond receptor recognition. In particular, the presence of CRISPR-Cas-related genes (e.g., *ygbF* encoding Cas2 and *casA*) in some EPIMRB01-adsorbing but resistant strains support the hypothesis that CRISPR-mediated immunity may limit EPIMRB01 replication after adsorption, contributing to its narrower effective host range.

Overall, this analysis suggests that, in addition to a shared genetic background influencing adsorption, specific outer membrane-associated genes such as *mlaA, ydcQ,* and ompD-2 may be linked to phage-specific adsorption phenotypes. These observations remain associative and should be validated experimentally. The findings also highlight the importance of considering bacterial defense repertoires when interpreting phage infectivity and host-range differences.

## 3. Discussion

The isolation and characterization of the lytic phages EPIMAM01 and EPIMRB01 from untreated wastewater sources in Beirut expand the currently limited collection of locally characterized phages active against MDR *E. coli.* These phages demonstrated rapid adsorption, efficient lytic activity, environmental stability, and distinct biofilm-disruption capabilities, each contributing unique therapeutic potentials.

Phage EPIMAM01 displayed superior lytic performance against planktonic *E. coli* cells even at MOI of 0.1, indicating high infectivity and robust replication dynamics. The higher estimated burst size observed for EPIMAM01 suggests it can produce more progeny per infected cell, an advantage for rapidly reducing bacterial populations [6]. Such properties are crucial for in vivo applications where phage distribution and local concentrations can be variable. In contrast, EPIMRB01, while slightly less effective against planktonic cells, demonstrated strong biofilm disruption capabilities, a major hurdle in treating chronic and device-associated infections [7]. The ability of EPIMRB01 to penetrate and reduce biofilm biomass suggests it may express depolymerases or other extracellular matrix-degrading enzymes, similar to phages vB_EcoM_ECO78 and vB_EcoP-EG1, which have shown potent biofilm disruption in *E. coli* [8,9]. Since biofilm-associated infections are notoriously difficult to eradicate due to the protective barrier they form against antibiotics and immune responses, EPIMRB01 represents a valuable tool, either alone or in combination with other phages or antimicrobials. The pH and thermal stability profiles of EPIMAM01 and EPIMRB01 further support their potential for therapeutic applications. Both maintained infectivity at physiologically relevant pH and temperatures, a feature also reported in other robust phages such as vB_EcoS_UTEC10 [6]. These stability profiles indicate favorable physicochemical robustness for downstream therapeutic research.

From a genomic standpoint, EPIMAM01 and EPIMRB01 share a strongly conserved T4-like genomic backbone, but ORF-level and conserved-domain analyses identified localized divergence in a terminal long-tail fiber-associated region. The proximal long-tail fiber proteins were highly conserved, whereas the distal long-tail fiber-associated sequence and adjacent downstream protein were substantially more divergent. CDD prediction detected a Phage_T4_gp36 domain in the distal long-tail fiber proteins of both phages and a Peptidase_S74 domain in both downstream proteins, while the additional predicted domain content of the downstream proteins differed. Because tail fiber-associated proteins participate in interactions between tailed phages and bacterial cells, this localized variation provides a plausible candidate genomic basis for differences in host interaction, bacteriolytic activity, and antibiofilm performance. However, the present analysis is computational and does not establish receptor specificity, a structural mechanism, or causality; these possibilities require experimental and, where appropriate, structural validation [10].

Host range analysis revealed that EPIMAM01 could infect a broader set of *E. coli* strains, suggesting a higher degree of receptor versatility or an ability to evade intracellular defense mechanisms, such as CRISPR-Cas systems. In contrast, EPIMRB01 showed more restricted replication, highlighting the impact of intracellular resistance mechanisms even when adsorption is successful. This finding is consistent with other studies demonstrating the crucial role of bacterial defense systems, including CRISPR-Cas and toxin-antitoxin modules, in determining phage infection outcomes [11,12]. Furthermore, the identification of specific outer membrane genes, such as *mlaA* and *ydcQ*, along with the outer membrane protein OmpD-2, as candidate adsorption-associated genes offers valuable targets for future engineering of phage specificity and for designing synthetic phages or receptor decoys to enhance therapeutic efficacy. Phage engineering approaches, including receptor-binding domain swapping and CRISPR-mediated genome editing, are promising directions to address narrow host range limitations and improve therapeutic precision [13,14].

This study has several limitations. The proposed links between bacterial defense-associated genes and phage susceptibility are based on comparative genomic associations and do not establish causality. Likewise, the candidate adsorption-associated genes identified in this study were not experimentally validated as phage receptors. In addition, all functional characterization was performed in vitro, and future work should evaluate these phages in vivo and through targeted mechanistic studies. Although sequence and conserved-domain analyses identified a plausible divergent terminal tail fiber-associated region, its contribution to phenotypic differences between EPIMAM01 and EPIMRB01 was not experimentally or structurally validated. Future studies could include resistant-mutant analysis, receptor identification, adsorption-blocking assays, tail fiber exchange experiments, and confidence-assessed structural modeling where appropriate.

In conclusion, EPIMAM01 and EPIMRB01 are two lytic *E. coli* phages with distinct but potentially complementary in vitro properties. EPIMAM01 showed stronger suppression of planktonic growth and a broader effective host range, whereas EPIMRB01 showed stronger activity against preformed biofilms. Genomic analysis supported their lytic character and highlighted candidate regions that may contribute to phenotypic differences. Future studies should validate the proposed adsorption-associated genes, clarify the contribution of bacterial defense systems, and evaluate the performance of these phages in vivo and in combination-based strategies.

## 4. Materials and Methods

### 4.1. Wastewater Sample Collection

Two wastewater samples were collected in 50 mL Falcon tubes from untreated wastewater sources in Ain El-Mreiseh and Ramlet El-Bayda, Beirut. Samples were centrifuged at 4000× *g* for 15 min, and the supernatants were filtered through 0.22 μm pore size disposable syringe filters to remove particulate debris.

### 4.2. Phage Isolation and Purification

Filtered wastewater samples were subjected to an enrichment step using *E. coli* ATCC 25922 as the host strain. Briefly, filtered samples were incubated with the host bacterium in Luria–Bertani (LB) broth at 37 °C with shaking. Following enrichment, cultures were centrifuged at 4000× *g* for 15 min and filtered through 0.22 μm syringe filters. The resulting filtrates were serially diluted in LB broth and mixed with *E. coli* ATCC 25922 adjusted to 0.5 McFarland (approximately 10^8^ CFU/mL), then plated using the double-layer agar (DLA) method to detect phage plaques [15]. Individual plaques were picked and suspended in SM buffer consisting of 50 mM Tris-HCl (pH 7.5), 100 mM NaCl, and 8 mM MgSO_4_. For propagation, each phage was amplified on an overnight culture of *E. coli* ATCC 25922 in LB broth at 37 °C with shaking. The resulting lysate was incubated for 18 h, centrifuged at 4000× *g* for 15 min, and filtered through a 0.22 μm syringe filter. Plaque purification by serial dilution and DLA plating was repeated for three consecutive rounds, with a single plaque selected at each round to ensure clonal purity [16].

### 4.3. Adsorption Rate Assay

The adsorption assay was performed to determine the rate at which free phage particles adsorbed to *E. coli* ATCC 25922 [17]. Briefly, 9 mL of bacterial suspension adjusted to 0.5 McFarland was mixed with 1 mL of phage lysate at an MOI of 10. Mixtures were incubated at room temperature, and 500 μL aliquots were collected at defined time points. Samples were centrifuged at 4000× *g* for 5 min, and the titer of unadsorbed phage particles remaining in the supernatant was determined by the DLA method. Adsorption efficiency was calculated from the reduction in free phage titer relative to time zero.

### 4.4. One-Step Growth Curve

One-step growth experiments were performed with minor modifications of a published protocol [18]. Briefly, 9 mL of *E. coli* ATCC 25922 adjusted to 0.5 McFarland was mixed with 1 mL of phage lysate at an MOI of 10 and incubated for 5 min at room temperature to allow adsorption. The mixture was then centrifuged at 4000× *g* for 5 min to remove unabsorbed phages, and the pellet was resuspended in 9 mL of LB broth. Samples were collected every 5 min, and phage titers were determined by the DLA method. The latent, rise, and plateau phases were determined from the resulting growth curves. Burst size was estimated based on the increase in phage titer between the end of the latent period and the plateau phase relative to the estimated number of infected cells at time zero, as inferred from the adsorption assay and initial bacterial concentration.

### 4.5. Bacteriolytic Assay

The bacteriolytic activities of EPIMAM01 and EPIMRB01 were evaluated at MOIs of 1 and 0.1 against *E. coli* ATCC 25922, as previously described with minor modification [19]. Each condition was tested in four replicates in a 96-well plate, together with a negative control containing LB broth only and a positive control containing LB broth and *E. coli* ATCC 25922. Bacterial growth was monitored by measuring optical density at 600 nm over 14 h at 37 °C with shaking.

### 4.6. Phage Host Range and Efficacy of Plating

Efficacy of plating (EOP) was calculated as the ratio of the phage titer (PFU/mL) obtained on each clinical isolate to the titer obtained on the reference host strain, *E. coli* ATCC 25922. To assess host range, 10 μL of tenfold serial dilutions of EPIMAM01 and EPIMRB01 were first tested by spot assay on bacterial lawns prepared from the clinical *E. coli* isolates and the reference host strain. Phage activity was recorded based on the presence or absence of visible lysis. For quantitative EOP determination, phage titers were then measured by plaque enumeration using the DLA method on susceptible strains [20]. The clinical *E. coli* isolates used in this study consisted of previously characterized AMR clinical strains with available whole-genome sequencing data obtained from the AUB Bacteriology biorepository. These isolates were previously described in Abdo Ahmad et al. (2025) [5]. 

### 4.7. pH and Thermal Stability Assays

The pH and thermal stabilities of EPIMAM01 and EPIMRB01 were evaluated by measuring residual phage titers after exposure to different conditions. For pH stability, phage suspensions were incubated in LB broth adjusted to pH 2–13 using HCl or NaOH and incubated at 37 °C for 18 h. For thermal stability, phage suspensions were incubated in pH 7 LB broth at 4, 22, 28, 37, 50, 60, and 70 °C for 3 h. Residual phage titers were determined in triplicate by the DLA method using *E. coli* ATCC 25922 as the host [21].

### 4.8. Biofilm Clearance Assay

Biofilm disruption was assessed in a 96-well plate using *E. coli* ATCC 25922. Overnight bacterial cultures grown in LB broth at 37 °C with shaking were adjusted to 0.5 McFarland and diluted 1:100 in fresh LB broth. Aliquots of 150 μL were added to wells for biofilm formation, while control wells received LB broth only. Plates were incubated for 18 h at 28 °C without shaking to allow biofilm formation. Planktonic cells were then removed, wells were washed with 1× PBS, and phage suspensions were added at an MOI of 1. EPIMAM01, EPIMRB01, and a non-*E. coli* phage control (Sal INF 122131 M phage) were tested. After 5 h of incubation at 28 °C, wells were washed again with 1× PBS, stained with 0.1% crystal violet, washed three times, and destained with 95% ethanol. Optical density was measured at 600 nm to quantify residual biofilm biomass [22,23]. Each condition was tested in at least five independent experiments.

### 4.9. DNA Extraction

Phage DNA was extracted using a modified phenol:chloroform:isoamyl alcohol protocol (Center for Phage Technology, College Station, TX, USA, 2018). Briefly, phage lysates were treated for 90 min at 37 °C with DNase I (20 mg/mL) and RNase A (10 mg/mL), followed by enzyme inactivation with 0.5 M EDTA. Capsids were disrupted with proteinase K (20 mg/mL) and 10% SDS at 56 °C. DNA was then extracted sequentially with phenol:chloroform:isoamyl alcohol (25:24:1) and chloroform:isoamyl alcohol (24:1), precipitated with 3 M sodium acetate and 100% ethanol at −20 °C, washed twice with 70% ethanol, and resuspended in nuclease-free water. DNA concentration was measured using a NanoDrop spectrophotometer (Waltham, MA, USA).

### 4.10. Phage Genome Sequencing and Bioinformatics Analysis

Phage DNA libraries were prepared using the Nextera library preparation kit (Illumina, San Diego, CA, USA) and sequenced as 2 × 250 bp reads on an Illumina MiSeq platform at the DNA Sequencing Facility of the American University of Beirut. Low-quality reads and adapter sequences were removed using Trimmomatic (Version 0.39) [24], and filtered reads were assembled using SPAdes v3.15.5 [25]. Assemblies yielded single-contig phage genomes with average sequencing depth greater than 300×. Coding sequences and tRNA genes were predicted and annotated using Pharokka (Version 1.8.0) [26]. Circular genome maps were generated using phagescope (Version 1.3) [27]. Additional screening for lysogeny-, virulence-, and antibiotic resistance-associated genes was performed using PhageScope [26]. Phage taxonomy and genomic relatedness were assessed using BLASTn 2.9.0+, VIRIDIC (Version 1.1) [28], and ViPTree (Version 4.0) [29]. To further characterize the major divergent region observed in the pairwise whole-genome alignment, GenBank annotations for EPIMAM01 and EPIMRB01 were used to identify ORFs located in the terminal long-tail fiber-associated region. Predicted protein sequences were extracted from the annotated CDS features, and corresponding ORFs were compared by global pairwise amino-acid alignment. Global amino-acid identity was calculated as the number of identical aligned residues divided by the total alignment length. The genomic organization of the region and pairwise identity values were visualized as Supplementary Figure S2, and ORF annotations, coordinates, protein IDs, and protein lengths were summarized in Supplementary Table S1.

### 4.11. In Silico Analysis of the Divergent Terminal Tail Fiber-Associated Region

To characterize the major divergent terminal region identified in the pairwise whole-genome alignment, GenBank annotations for EPIMAM01 (PQ493298.1) and EPIMRB01 (PQ657784.1) were used to identify ORFs in the terminal long-tail fiber-associated region. Predicted protein sequences were extracted from annotated CDS features, and corresponding proteins were compared using global pairwise amino-acid alignment. Global amino-acid identity was calculated as the number of identical aligned residues divided by the total alignment length. For conserved-domain prediction, the four proteins in the most divergent segment (EPIMAM01_00250, EPIMAM01_00251, EPIMRB01_00250, and EPIMRB01_00252) were analyzed using NCBI Batch CD-Search against the Conserved Domain Database (CDD) [30], with an E-value threshold of 0.01. ORF organization, protein identity values, and predicted domain features were visualized in Supplementary Figure S2 and summarized in Supplementary Tables S1 and S2. No three-dimensional structural modeling was performed.

### 4.12. Bacterial Pangenome Analysis

Bacterial genomes used in this study were obtained from pathogenic isolates stored in the bacteriology laboratory biorepository at the AUB Medical Center. A total of 18 genomes were included in the pangenome analysis, which was performed using Roary v3.11.2 [31]. A phylogenetic tree based on the core genome alignment was generated from genes shared by all analyzed strains and visualized using iTOL v6.0 [32]

### 4.13. Analysis of Phage Resistance Mechanisms

Candidate bacterial defense mechanisms, including CRISPR-Cas systems, restriction–modification systems, and toxin–antitoxin modules, were identified from previously generated whole-genome sequencing data. Genomic DNA extraction and sequencing had been performed previously using the Qiagen DNeasy kit and the Illumina NovaSeq 6000 platform (San Diego, CA, USA) (150 bp paired-end reads). Assemblies were generated with SPAdes v3.15.3. and annotated with Prokka v1.8.0 [1].

### 4.14. Statistical Analysis

Statistical analyses and figure generation were performed in Python 3.12.0. Biofilm data were analyzed using one-way ANOVA followed by a post hoc multiple-comparison test, with *p* < 0.05 considered statistically significant. To evaluate associations between gene presence and phage infection outcome, contingency-table analyses were performed for each gene. Correlation strength was summarized using Cramér’s V statistic. The −log_10_(*p*-value) was used for visualization of association strength. For sparse contingency tables, Fisher’s exact test was used. *p*-values from gene-level analyses were adjusted for multiple testing using the Benjamini–Hochberg procedure.

## Data Availability

The original contributions presented in this study are included in the article; further inquiries can be directed to the corresponding author.

## Supplementary Materials

The following supporting information can be downloaded at: https://www.mdpi.com/article/10.3390/antibiotics15060563/s1. Figure S1: VIRIDIC-based intergenomic similarity analysis of EPIMRB01 and EPIMAM01. Figure S2: Sequence and conserved-domain analysis of the divergent terminal long-tail fiber-associated region in EPIMAM01 and EPIMRB01. Table S1: ORF-level comparison of the divergent terminal long-tail fiber-associated region in EPIMAM01 and EPIMRB01. Table S2: Conserved-domain prediction for proteins in the most divergent terminal tail fiber-associated segment of EPIMAM01 and EPIMRB01.
