# Supplementary material for: Comparative Genomic and Functional Characterization of Two Lytic Bacteriophages Against Antimicrobial-Resistant Escherichia coli
Source: Antibiotics (Basel). 2026 Jun 1;15(6):563. doi: 10.3390/antibiotics15060563 (PMC13295566; doi:10.3390/antibiotics15060563)
Supplement: Supplementary file 1 [file antibiotics-15-00563-s001.zip › Supplementary Figure S1.pdf]

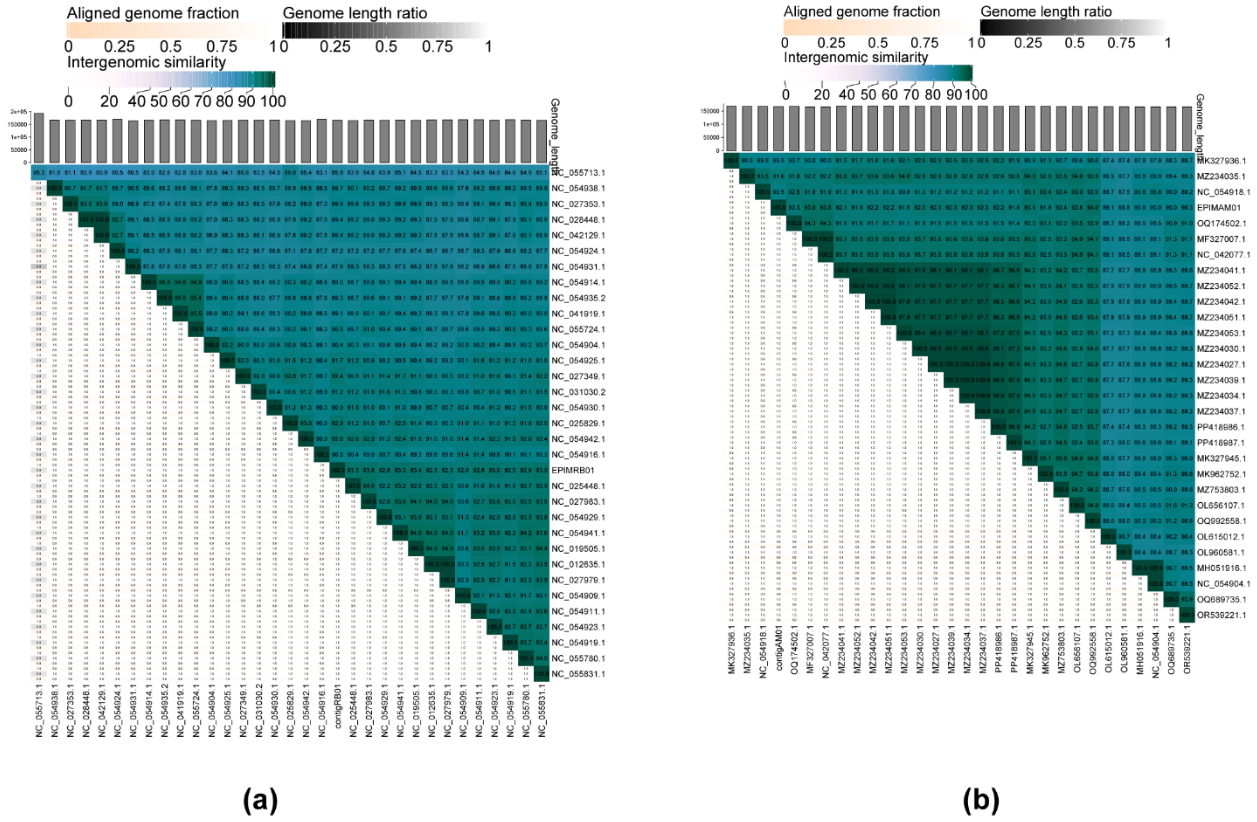

Supplementary Figure S1: VIRIDIC-based intergenomic similarity analysis of EPIMRB01 and EPIMAM01. Heat maps generated using the Virus Intergenomic Distance Calculator (VIRIDIC) showing pairwise nucleotide similarity between the newly isolated phages EPIMRB01 (a) and EPIMAM01 (b) and their closest related reference phages. Each cell represents the percentage of intergenomic similarity between phage genomes, with color intensity corresponding to the degree of nucleotide similarity. The 95% intergenomic similarity threshold was included as a reference for species-level demarcation.
