# Supplementary material for: Comparative Genomic and Functional Characterization of Two Lytic Bacteriophages Against Antimicrobial-Resistant Escherichia coli
Source: Antibiotics (Basel). 2026 Jun 1;15(6):563. doi: 10.3390/antibiotics15060563 (PMC13295566; doi:10.3390/antibiotics15060563)
Supplement: Supplementary file 1 [file antibiotics-15-00563-s001.zip › Supplementary Figure S2.pdf]

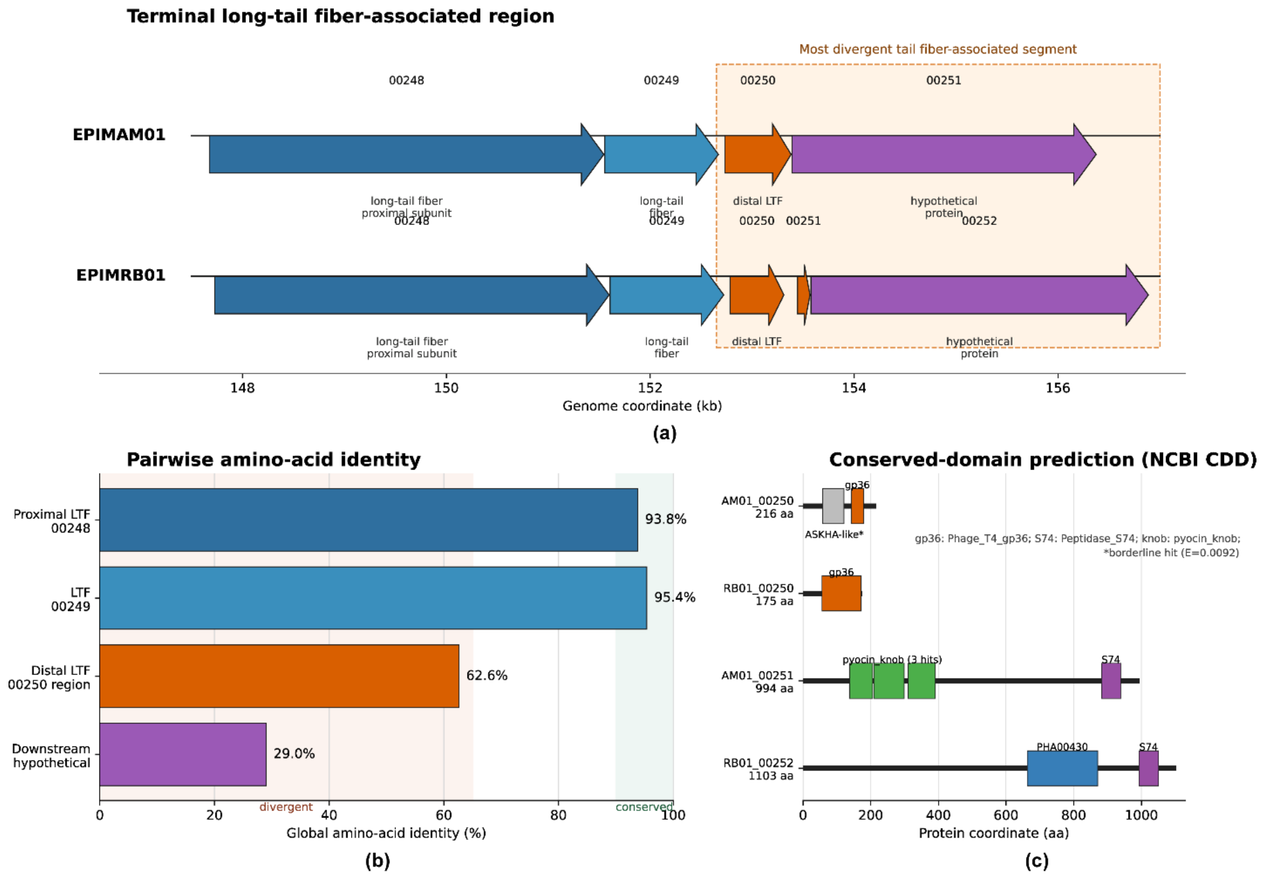

Supplementary Figure S2: Sequence and conserved-domain analysis of the divergent terminal long-tail fiber-associated region in EPIMAM01 and EPIMRB01. (a) Genomic organization of the terminal long-tail fiber-associated region based on GenBank annotations. (b) Global pairwise amino-acid identity values for corresponding proteins; the proximal long-tail fiber proteins were highly conserved, whereas the distal long-tail fiber-associated region and adjacent downstream protein were more divergent. For the split EPIMRB01 distal long-tail fiber annotation, EPIMRB01_00250 and EPIMRB01_00251 were considered together for sequence comparison with EPIMAM01_00250. (c) NCBI CDD conserved-domain predictions for proteins in the most divergent segment. Phage_T4_gp36 and Peptidase_S74 features were detected in proteins from both phages, whereas additional predicted domain content differed between the downstream proteins. These computational findings support localized divergence in a candidate tail fiber-associated region but do not establish its functional role.
