# Supplementary material for: Comparative Genomic and Functional Characterization of Two Lytic Bacteriophages Against Antimicrobial-Resistant Escherichia coli
Source: Antibiotics (Basel). 2026 Jun 1;15(6):563. doi: 10.3390/antibiotics15060563 (PMC13295566; doi:10.3390/antibiotics15060563)
Supplement: Supplementary file 1 [file antibiotics-15-00563-s001.zip › Supplementary Table S1.pdf]

Supplementary Table S1: ORF-level comparison of the divergent terminal long-tail fiber-associated region in EPIMAM01 and EPIMRB01. Coordinates, product annotations, protein IDs, and predicted protein lengths were extracted from the GenBank annotations of EPIMAM01 (PQ493298.1) and EPIMRB01 (PQ657784.1).

Phage	Accession	Locus tag	Coordinates	Strand	Product annotation	Protein ID	Protein length (aa)
EPIMAM01	PQ493298.1	EPIMAM01_00248	147676..151545	+	long-tail fiber proximal subunit	XMR90270.1	1289
EPIMAM01	PQ493298.1	EPIMAM01_00249	151554..152669	+	long-tail fiber protein	XMR90271.1	371
EPIMAM01	PQ493298.1	EPIMAM01_00250	152732..153382	+	long-tail fiber protein	XMR90272.1	216
EPIMAM01	PQ493298.1	EPIMAM01_00251	153391..156375	+	hypothetical protein	XMR90273.1	994
EPIMRB01	PQ657784.1	EPIMRB01_00248	147728..151597	+	long-tail fiber proximal subunit	XLL18578.1	1289
EPIMRB01	PQ657784.1	EPIMRB01_00249	151606..152721	+	long-tail fiber protein	XLL18579.1	371
EPIMRB01	PQ657784.1	EPIMRB01_00250	152784..153311	+	long-tail fiber protein	XLL18580.1	175
EPIMRB01	PQ657784.1	EPIMRB01_00251	153445..153567	+	long-tail fiber protein	XLL18581.1	40
EPIMRB01	PQ657784.1	EPIMRB01_00252	153576..156887	+	hypothetical protein	XLL18582.1	1103
