# Supplementary material for: Comparative Genomic and Functional Characterization of Two Lytic Bacteriophages Against Antimicrobial-Resistant Escherichia coli
Source: Antibiotics (Basel). 2026 Jun 1;15(6):563. doi: 10.3390/antibiotics15060563 (PMC13295566; doi:10.3390/antibiotics15060563)
Supplement: Supplementary file 1 [file antibiotics-15-00563-s001.zip › Supplementary Table S2.pdf]

Supplementary Table S2: Conserved-domain prediction for proteins in the most divergent terminal tail fiber-associated segment of EPIMAM01 and EPIMRB01. Protein sequences were analyzed using NCBI Batch CD-Search against CDD with an E-value threshold of 0.01. Reported hits should be interpreted as computational predictions and not as experimental functional assignments.

Phage	Locus tag	Protein ID	Product annotation	CDD hit type	Domain accession	Domain name	Protein coordinates (aa)	E-value	Interpretation
EPIMAM01	EPIMAM01_00250	XMR90272.1	long-tail fiber protein	non-specific	pfam03903	Phage_T4_gp36	143..178	0.00102954	Distal tail-fiber-associated domain
EPIMAM01	EPIMAM01_00250	XMR90272.1	long-tail fiber protein	non-specific	cd24110	ASKHA_NBD_NTPDase1	58..120	0.0092294	Borderline CDD hit; interpret cautiously
EPIMAM01	EPIMAM01_00251	XMR90273.1	hypothetical protein	specific	pfam13884	Peptidase_S74	882..938	1.71E-12	Peptidase_S74-associated domain
EPIMAM01	EPIMAM01_00251	XMR90273.1	hypothetical protein	specific	cd19958	pyocin_knob	137..205	1.67E-06	Predicted receptor-binding/knob-associated repeat
EPIMAM01	EPIMAM01_00251	XMR90273.1	hypothetical protein	specific	cd19958	pyocin_knob	209..298	2.73E-10	Predicted receptor-binding/knob-associated repeat
EPIMAM01	EPIMAM01_00251	XMR90273.1	hypothetical protein	specific	cd19958	pyocin_knob	310..390	3.39E-07	Predicted receptor-binding/knob-associated repeat
EPIMRB01	EPIMRB01_00250	XLL18580.1	long-tail fiber protein	non-specific	pfam03903	Phage_T4_gp36	56..171	1.27E-19	Distal tail-fiber-associated domain
EPIMRB01	EPIMRB01_00252	XLL18582.1	hypothetical protein	specific	pfam13884	Peptidase_S74	993..1050	2.28E-12	Peptidase_S74-associated domain
EPIMRB01	EPIMRB01_00252	XLL18582.1	hypothetical protein	non-specific	PHA00430	PHA00430	663..870	8.38E-06	Phage-associated predicted domain; function not assigned here
